# Outcomes After Distal Pancreatectomy with Celiac Axis Resection for Pancreatic Cancer: A Pan-European Retrospective Cohort Study

**DOI:** 10.1245/s10434-018-6391-z

**Published:** 2018-03-12

**Authors:** Sjors Klompmaker, Jony van Hilst, Sarah L. Gerritsen, Mustapha Adham, M. Teresa Albiol Quer, Claudio Bassi, Frederik Berrevoet, Ugo Boggi, Olivier R. Busch, Manuela Cesaretti, Raffaele Dalla Valle, Benjamin Darnis, Matteo De Pastena, Marco Del Chiaro, Robert Grützmann, Markus K. Diener, Traian Dumitrascu, Helmut Friess, Arpad Ivanecz, Anastasios Karayiannakis, Giuseppe K. Fusai, Knut J. Labori, Carlo Lombardo, Santiago López-Ben, Jean-Yves Mabrut, Willem Niesen, Fernando Pardo, Julie Perinel, Irinel Popescu, Geert Roeyen, Alain Sauvanet, Raj Prasad, Christian Sturesson, Mickael Lesurtel, Jorg Kleeff, Roberto Salvia, Marc G. Besselink, the E-AHPBA DP-CAR study group, Panagis Lykoudis, Thilo H. Hackert, Zeeshan Ateeb

**Affiliations:** 1 Department of Surgery, Cancer Center Amsterdam, Academic Medical Center, Amsterdam, the Netherlands; 2 Department of Digestive Surgery, E. Herriot Hospital, HCL, UCBL1, Lyon, France; 3 Department of Surgery, Hospital Universitari de Girona Dr. Josep Trueta, Girona, Spain; 4 Department of Surgery, University of Verona, Verona, Italy; 5 Department of General and HPB Surgery, Ghent University Hospital, Ghent, Belgium; 6 Division of General and Transplant Surgery, University of Pisa, Pisa, Italy; 7 Department of HPB Surgery, Hôpital Beaujon, Clichy Cedex, France; 8 Hepato-Pancreato-Biliary Unit, Parma University Hospital, Parma, Italy; 9 Department of Surgery and Liver Transplantation, Croix-Rousse University Hospital, Hospices Civils de Lyon, University of Lyon I, Lyon, France; 10 Department of Clinical Science, Intervention and Technology, Karolinska University Hospital, Stockholm, Sweden; 11 Department of Surgery, University Hospital Erlangen, Erlangen, Germany; 12 Department of General, Visceral and Transplantation Surgery, Heidelberg University, Heidelberg, Germany; 13 Center of General Surgery and Liver Transplant, Fundeni Clinical Institute, Bucharest, Romania; 14 Department of Surgery, Klinikum rechts der Isar, School of Medicine, Technical University of Munich, Munich, Germany; 15 Department of Abdominal and General Surgery, University Medical Centre Maribor, Maribor, Slovenia; 16 Second Department of Surgery, Democritus University of Thrace, Alexandroupolis, Greece; 17 HPB Surgery and Liver Transplantation Unit, Royal Free Hospital, London, UK; 18 Department of Hepato-Pancreato-Biliary Surgery, Oslo University Hospital, Oslo, Norway; 19 Department of HPB and Transplant Surgery, Clínica Universidad de Navarra, Pamplona, Spain; 20 Department of Hepatobiliary, Endocrine and Transplantation Surgery, Antwerp University Hospital, Antwerp, Belgium; 21 Department of HPB and Transplant Services, National Health Service, Leeds, UK; 22 Department of Surgery, Skåne University Hospital, Lund, Sweden; 23 Department of Visceral, Vascular and Endocrine Surgery, Martin-Luther-University Halle-Wittenberg, Halle, Germany

## Abstract

**Background:**

Western multicenter studies on distal pancreatectomy with celiac axis resection (DP-CAR), also known as the Appleby procedure, for locally advanced pancreatic cancer are lacking. We aimed to study overall survival, morbidity, mortality and the impact of preoperative hepatic artery embolization (PHAE).

**Methods:**

Retrospective cohort study within the European-African Hepato-Pancreato-Biliary-Association, on DP-CAR between 1-1-2000 and 6-1-2016. Primary endpoint was overall survival. Secondary endpoints were radicality (R0-resection), 90-day mortality, major morbidity, and pancreatic fistulae (grade B/C).

**Results:**

We included 68 patients from 20 hospitals in 12 countries. Postoperatively, 53% of patients had R0-resection, 25% major morbidity, 21% an ISGPS grade B/C pancreatic fistula, and 16% mortality. In total, 82% received (neo-)adjuvant chemotherapy and median overall survival in 62 patients with pancreatic ductal adenocarcinoma patients was 18 months (CI 10–37). We observed no impact of PHAE on ischemic complications.

**Conclusions:**

DP-CAR combined with chemotherapy for locally advanced pancreatic cancer is associated with acceptable overall survival. The 90-day mortality is too high and should be reduced. Future studies should investigate to what extent increasing surgical volume or better patient selection can improve outcomes.

**Electronic supplementary material:**

The online version of this article (10.1245/s10434-018-6391-z) contains supplementary material, which is available to authorized users.

Locally advanced pancreatic cancer has a median survival ranging from 6 to 24 months, depending on the ability to undergo both local and systemic treatment.^1^^–^3 In selected cases, distal pancreatectomy with celiac axis resection (DP-CAR) can lead to radical tumor removal in otherwise borderline or unresectable disease.^4^^–^13 After celiac axis resection, retrograde flow from the superior mesenteric artery via the pancreatoduodenal arcades feeds the pancreatic head and the liver.^14^ In addition, some centers apply preoperative hepatic artery embolization (PHAE) in an attempt to improve collateral flow and reduce postoperative (liver) ischemia, although its impact remains unclear.^14^,^15^

In a recent systematic review, we have shown that a highly selected group of patients may benefit from DP-CAR. In an analysis of 240 patients, overall survival was 18 months when DP-CAR was combined with (neo-)adjuvant chemotherapy at an acceptable 90-day mortality rate of 3.5%.^14^ However, only relatively small studies (median 7 patients) of low-to-moderate quality could be included, covering a 40-year period. The recent uptake of neoadjuvant FOLFIRINOX (folinic acid, fluorouracil, irinotecan, oxaliplatin) may eventually lead to higher down-staging rates for pancreatic cancer, which could increase the application of DP-CAR and improve survival.^3^,^16^

More recent reports, originating from the United States and Japan, showed short-term mortality rates between 5 and 14% and median overall survival ranged from 17 to 40 months.^17^^–^20 However, still only single-center studies exist, with the largest Western series consisting of 30 patients.^17^ The purpose of this pan-European study was to assess overall survival and complications after DP-CAR, including the effect of chemotherapy and PHAE, in a relatively large, multicenter cohort.

## Methods

We performed a pan-European retrospective single-arm cohort study on DP-CAR, among centers represented by members of the European-African Hepato-Pancreato-Biliary Association (E-AHPBA). The study protocol, including an analysis framework, was initiated and approved by the E-AHPBA research and scientific committee and made available online.^21^ We invited all E-AHPBA members who had performed DP-CAR between January 1, 2000 and May 31, 2016 to participate. The institutional review board at the Academic Medical Center Amsterdam waived the need for ethical review.

### Patients and Data Collection

All participating centers completed an online survey (Google™ Survey, Mountain View, CA) containing questions regarding standards of care and annual volumes for pancreatic surgery. Each center appointed a local study coordinator, responsible for questionnaire completion and data collection. Subsequently, we retrieved all consecutive patients who underwent DP-CAR for pancreatic cancer within the study period. Patients were excluded in case of non-pancreatic carcinoma diagnosis. Each center submitted baseline (sex, age, BMI, ASA classification, surgical history, and tumor characteristics), treatment (neoadjuvant therapy, embolization, operative variables, adjuvant therapy), and outcome data (morbidity, mortality, length of stay, histopathology, and survival) anonymously using predefined online case report forms (CRF). All data were collected and analyzed by the central study coordinators (SK and JH).

### Definitions

American Joint Committee on Cancer (AJCC) stage, tumor size, and additional organ and vascular involvement (other than pancreas, spleen, celiac axis, or splenic vessels) were based on preoperative imaging (CT or MRI) and postoperative pathology reports.^22^ Pre- and postoperative chemotherapy and radiotherapy treatment was recorded, including the use of FOLFIRINOX. PHAE was defined by preoperative intraluminal catheter embolization of the common hepatic artery. The intention to perform DP-CAR versus intraoperative conversion from distal pancreatectomy to DP-CAR was recorded in a separate variable (intended vs. nonintended).

Postoperative complications were scored as major morbidity (grade 3a–4b) based on the Clavien-Dindo classification of surgical complications.^23^ The definitions of the International Study Group on Pancreatic Surgery (ISGPS) were used to score postoperative pancreatic fistula, delayed gastric emptying, and post-pancreatectomy hemorrhage.^24^^–^26 Surgical site infection was defined using the Center for Disease Control and Prevention (CDC) definitions.^27^ Ischemic morbidity was defined as an abdominal organ complication caused by surgery-related ischemia.

Resection margins, including transection and circumferential margins, were categorized according to the Royal College of Pathologists definition and were classified as R0 (no residual, distance margin to tumor ≥ 1 mm), R1 (residual tumor, distance margin to tumor < 1 mm), and R2 (residual tumor, macroscopically positive margin).^28^ Complications, readmissions, and mortality were all collected up to 90 days postoperatively. Overall survival was collected based on the last visit to the hospital, follow-up phone calls, or national security registries depending on the country of origin.

### Outcomes

Primary outcome was overall survival. Secondary outcomes were R0 resection margin, lymph node harvest, postoperative mortality, morbidity (including ischemic (liver) morbidity, postoperative pancreatic fistula, delayed gastric emptying, post-pancreatectomy hemorrhage, organ space (abdominal) infection), reinterventions, length of hospital stay, and readmissions.

### Statistical Analysis

All statistical analyses were performed using STATA version 14.1 IC (StataCorp LP, College Station, TX). Categorical data are presented as counts and proportions. Continuous data are presented as both mean (standard deviation) and median (interquartile range). All confidence intervals (CI) are 95%, and alpha levels for significance are < 0.050. The Mann–Whitney *U* test and Fisher’s exact test were used to compare continuous or categorical data, respectively. We used Kaplan–Meier curves, stratified by (neo-)adjuvant therapy regimen, to assess overall survival after DP-CAR. We used the log-rank test to determine significant differences in survival. To assess the impact of annual pancreatic surgery case volume, we performed a sensitivity analysis wherein we excluded all centers at or below the median case volume for pancreatoduodenectomy. We performed a univariate screen (*P* < 0.20) and multivariable analysis to assess potential factors associated with 90-day mortality.

## Results

Of 35 initial responding hospitals, 20 hospitals across 12 European countries fulfilled the eligibility criteria and included 72 patients undergoing DP-CAR between January 1, 2000 and May 31, 2016. After exclusion of three neuroendocrine tumors and one non-Hodgkin lymphoma, 68 patients with exocrine pancreatic cancer remained. All participating hospitals were high-volume pancreatic centers (median of 70 pancreatoduodenectomies [interquartile range (IQR) 31–88] per year). The median total case volume for DP-CAR was 3 (IQR 2–5). Of the participating centers, 14 (70%) reported using DP-CAR in case of intraoperatively detected celiac axis tumor involvement and 3 (15%) reported routine use of PHAE.

### Baseline and Treatment

Baseline characteristics are described in Table 1. Preoperatively, 15 (22%) patients received neoadjuvant chemotherapy, 19 (28%) patients received neoadjuvant chemoradiotherapy, and 15 (22%) patients received PHAE. A minimally invasive DP-CAR was performed in 2 (2.9%) patients. Vascular resection was performed in 18 (27%) patients and adrenal gland resection in 15 (22%) patients. A total of 9 (13%) patients underwent hepatic artery reconstruction because of insufficient collateral flow via the pancreatoduodenal arcade (Table 2). This included aortae to hepatic artery (*n* = 6), superior mesenteric to hepatic artery (*n* = 2), and gastroduodenal to hepatic artery confluence (*n* = 1) bypasses.

**Table 1:** Baseline characteristics

	(*N* = 68)
Baseline	
Female sex, no. (%)	32 (47.1)
Age, median (IQR), year	60 (52–67)
Mean (SD), year	58.9 (10.6)
Body-Mass-Index, median (IQR), kg/m^2^	24 (22–26.5)
Mean (SD), kg/m^2^	24.3 (3.6)
ASA-classification, no. (%)	
ASA-1	12 (17.7)
ASA-2	50 (73.5)
ASA-3	6 (8.8)
Abdominal surgery history ≥ 1, no. (%)	21 (32.8)
Preoperative tumor characteristics	
Additional organ involvement*, no. (%)	
Stomach	6 (8.8)
Liver	1 (1.5)
Kidney	3 (4.4)
Adrenal gland	5 (7.4)
Additional vascular involvement, no. (%)	
Hepatic artery	8 (11.8)
Superior mesenteric artery	7 (10.3)
Portal vein	6 (8.8)
Superior mesenteric vein	9 (13.2)
Preoperative tumor size, median (IQR), mm	37 (30–50)
Mean (SD), mm	43 (33)
AJCC staging**, no. (%)	
T-stage ≥ 3	62 (95.4)
N-stage > 0	20 (29.9)
M-stage > 0	1 (1.5)

*ASA* American Society of Anesthesiologists

*Other than celiac axis, pancreas, or spleen

**Based on the AJCC criteria^22^

**Table 2:** Treatment characteristics

	(*N* = 68)
Preoperative	
Neoadjuvant treatment, no. (%)	
Chemotherapy	15 (22.1)
Chemoradiotherapy	19 (27.9)
Preoperative hepatic artery embolization, no. (%)	15 (22.1)
Operative	
Intent to perform DP-CAR	55 (80.9)
Operative time, median (IQR), min	328 (244–415)
Mean (SD), min	341 (124)
Additional organs resected*, no. (%)	
Stomach	7 (10.3)
Liver	3 (4.4)
Kidney	3 (4.4)
Adrenal gland	15 (22.1)
Additional vessels resected, no. (%)	
Right/left hepatic artery	1 (1.5)
Superior mesenteric artery	1 (1.5)
Portal vein	6 (8.8)
Superior mesenteric vein	10 (14.7)
Vascular reconstruction, no. (%)	
Common hepatic artery	9 (13.2)
Superior mesenteric artery	1 (1.5)
Portal vein	6 (8.8)
Superior mesenteric vein	3 (4.4)
Estimated blood loss, median (IQR), mL	500 (350–1300)
Mean (SD), mL	922 (893)
Blood transfusion for bleeding (< 72 h), no. (%)	20 (31.3)
Postoperative	
Adjuvant treatment, no. (%)	
Chemotherapy	41 (60.3)
Radiotherapy	2 (2.9)
Chemoradiotherapy	2 (2.9)

*Other than celiac axis, pancreas, or spleen

### Short-term Outcomes

R0 resection was achieved in 36 (55%) cases, with a median lymph node harvest of 22 (IQR 16–30). After surgery, 7 (10%) patients died within 30-days and 11 (16%) patients died within 90 days, all due to complications. Causes of death were related to gastric ischemia (*n* = 3), liver ischemia (*n* = 2), post-pancreatectomy hemorrhage (*n* = 2), pneumonia (*n* = 2), abdominal infection (*n* = 1), and sepsis with multi-organ failure (*n* = 1). Major morbidity occurred in 17 (25%) patients and an ISGPF grade B/C fistula in 14 (21%) patients. Median length of stay was 17 (IQR 11–27) days, with readmission in 9 (14%) patients (Table 3). Between patients who did (*n* = 15) and did not (*n* = 53) receive PHAE, we found similar rates of liver ischemia (19% vs. 20%, *P* > 0.99) and 90-day mortality (11% vs. 17%, *P* > 0.99). Reoperations were performed in 10 (14.7%) patients. Reoperations were gastric (wedge) resection for ischemia (*n* = 3), hepatic artery hemorrhage repair (*n* = 2), re-do anastomosis for a hepatic confluence thrombus (*n* = 1) or hemorrhage (*n* = 1), gastrojejunostomy for persistent delayed gastric emptying (*n* = 1), right hemicolectomy for a perforation (*n* = 1), and embolectomy of the right popliteal artery (*n* = 1).

**Table 3:** Ninety-day outcomes after DP-CAR

	(*N* = 68)
Outcomes	
Mortality within 30 days, no. (%)	7 (10.3)
Mortality within 90 days, no. (%)	11 (16.4)
Complications within 90 days, no. (%)	
Clavien-Dindo 3a–4b	17 (25)
Post-pancreatectomy hemorrhage*, no. (%)	6 (8.8)
Liver ischemia	12 (17.7)
Abdominal cavity infection, no. (%)	4 (5.9)
Pancreatic fistula grade B/C* no. (%)	14 (20.6)
Delayed gastric emptying grade B/C*, no. (%)	11 (17.5)
Reinterventions, no. (%)	
Endoscopic intervention, no. (%)	1 (1.6)
Radiologic drainage, no. (%)	9 (14.5)
Reoperation, no. (%)	10 (14.7)
Gastric (wedge) resection for ischemia	3
Hemorrhage repair	2
Re-do vascular anastomosis	2
Gastrojejunostomy for DGE	1
Repair of metastatic colon perforation	1
Peripheral arterial embolectomy	1
Histopathology	
Malignant etiology, no. (%)	
PDAC	62 (91.2)
Invasive IPMN	3 (4.4)
Other malignant diagnosis	3 (4.4)
Tumor size, median (IQR), mm	40 (32–50)
Mean (SD), mm	44 (23)
Resection margin, no. (%)	
R0	36 (54.6)
R1	28 (42.4)
R2	2 (3)
Lymph nodes harvested, median (IQR), no.	22 (16–29.5)
Median (SD), no.	25 (15)
Lymph node metastasis, no. (%)	45 (66.2)
Length of hospital say, median (IQR), days	16.5 (11–27)
Mean (SD), days	20 (14)
Unplanned readmission, no. (%)	9 (13.9)
Overall survival, median (CI), months	17 (10–33)

*IPMN* intraductal papillary mucinous neoplasm, *PDAC* pancreatic ductal adenocarcinoma

*ISGPS definitions^24^^**–**^26

### Survival

**Fig. 1:**
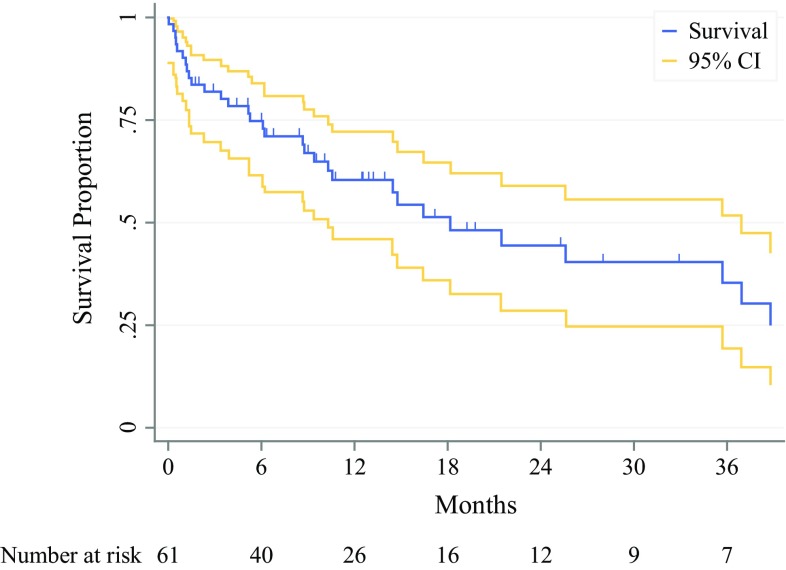
Survival curve after DP-CAR for pancreatic ductal adenocarcinoma. Kaplan–Meier survival estimate for 62 patients with pancreatic ductal adenocarcinoma, excluding three patients with invasive IPMN, and three patients with atypical pancreatic carcinomas. Median overall survival was 18 (CI 10–37) months. Vertical bars indicate censored cases and yellow lines indicate the 95% confidence interval (CI)

Postoperative follow-up time ranged from 0 to 66 months, with a median of 10 months (IQR 4–19). During the follow-up, 40 (59%) patients expired. This was assessed by means of follow-up phone calls (49%), medical record review (41%), or trough social security registry review (10%). Of all patients, 56 (82%) received either neoadjuvant or adjuvant chemotherapy, of which 12 (18%) received at least one cycle of FOLFIRINOX (neoadjuvant and adjuvant therapy characteristics; Supplement 1). Among the 62 patients with pancreatic ductal adenocarcinoma, Kaplan–Meier estimated median overall survival was 18 months (CI 10–37) (Fig. 1). In this group, 1-year survival was 60% (CI 46–72%) and 2-year survival was 45% (CI 29–59%).

### Sensitivity and Subgroup Analysis

The sensitivity analysis indicated a nonsignificant trend towards lower 90-day mortality in centers with an annual pancreatoduodenectomy case volume above the median (70 per year), total DP-CAR volume above 5, and procedure year after 2008 (see Supplement 2). Among all 68 patients, exploratory sub group analyses assessed neoadjuvant and/or adjuvant chemotherapy/chemoradiation versus no (neo-)adjuvant therapy (Supplement 3a), neoadjuvant versus no neoadjuvant chemotherapy/chemoradiation (Supplement 3b), and adjuvant versus no adjuvant chemotherapy/chemoradiation (Supplement 3c). However, the sample sizes became too low to achieve real statistical solidity.

### Predicting 90-day Mortality

Univariable analysis indicated potential predictors for 90-day mortality: mortality and male sex, additional vascular involvement on CT/MRI, no neoadjuvant chemotherapy, nonintended versus intended DP-CAR, and an annual pancreatoduodenectomy volume above the mean. However, except for male sex (odds ratio [OR] 9.45, *P* = 0.04), none of these remained significant in multivariable analysis (Supplement 4).

## Discussion

In this largest Western series on DP-CAR to date, we found a median overall survival of 18 months in 62 patients with pancreatic ductal adenocarcinoma involving the celiac axis. We observed considerable 30-day (10%) and 90-day (16%) mortality, without evidence indicating a beneficial effect of PHAE on the need for arterial reconstruction or ischemic complications. We observed a nonsignificant trend for reduced risk of 90-day mortality among high-volume centers.

These survival and morbidity outcomes are comparable to prior evidence, although the 90-day mortality rate was high. Overall survival after DP-CAR in the literature ranges from median 17–20 months in two recent smaller (*n* < 20) series and one systematic review (*n* = 240) by our group to median 31–35 months in two larger series (*n* > 25) from Sapporo and Pittsburgh.^14^,^17^^–^20 Overall survival for unresected patients with locally advanced pancreatic cancer (AJCC Stage III) ranges from 7 months in a large population-based study (*n* = 12,981) to 16–21 months with FOLFIRINOX in single-center studies (*n* = 46–70).^1^,^29^,^30^ However, the existing evidence lacks the necessary detailed information to study vascular involvement.

Postoperative mortality rates in the literature range from 5% (4 of 80 patients) in-hospital mortality in the Sapporo cohort to 14% (4 of 30 patients) 90-day mortality in the Pittsburgh cohort.^17^,^19^ The latter included 11 patients who underwent robot-assisted DP-CAR with 0% 90-day mortality.^17^ Major morbidity rates in the published literature range from 10% to more than 25%, but definitions are heterogeneous.^14^,^17^^–^19 The R1 rate (43%) and lymph node positive rate (66%) were comparable to the results from the recent ESPAC-4 trial.^31^ Reports on PHAE in the literature remain scarce, with routine use primarily reported by Japanese studies.^14^

Although our study showed no evidence that PHAE leads to fewer ischemic complications, no final conclusions can be drawn. Apart from a lack of power to detect smaller effects, PHAE may have prevented some aborted surgeries when insufficient collateral flow was found before surgery. Moreover, we were unable to study the potentially beneficial effects of embolization of all three celiac axis branches versus the common hepatic artery alone, as described by Cesaretti and colleagues.^32^ We also could not assess the impact of preservation or reconstruction of the left gastric artery using the middle colic artery on gastric ischemia, as described by Okada and colleagues.^33^ Such techniques can only be adequately studied via prospective registries, such as the Arterial Network, including patients in whom intended DP-CAR was aborted because of insufficient collateral blood flow.^34^ Conversely, we found that in 13 (20%) patients, DP-CAR was performed as an extension to distal pancreatectomy in which initially no vascular resection was planned.

In contrast to our expectations, we did not find a significant association between neoadjuvant chemotherapy and improved survival after DP-CAR. However, the recent report from Pittsburgh (*n* = 30), in which the authors describe a 96% neoadjuvant therapy rate and a 35-month median overall survival, suggests an important role for neoadjuvant treatment.^17^ As the authors state, neoadjuvant therapy can be given to downstage the tumor but more importantly to enable detection and treatment of occult micrometastatic disease before committing patients to DP-CAR.^17^ Now that FOLFIRINOX treatment has become the new standard of care, the benefit of neoadjuvant chemotherapy may increase further.^17^,^18^,^20^ The assessment of vascular involvement on imaging after FOLFIRINOX in pancreatic cancer is unreliable.^35^ In our study, seven patients appeared to have SMA involvement, whereas only one patient required a SMA resection.

This study had several limitations. First, we were unable to include a control group, because a comparable sample of unresected patients with celiac axis involvement was unavailable. Second, selection or reporting bias may have occurred through self-selection by centers with favorable experience with DP-CAR. We aimed to limit this effect by giving anonymity to participating centers. Third, although we tried to collect the biggest Western sample to date, our sample size remains limited. Fourth, study design and data collection commenced before the release of the 8th edition of the AJCC staging criteria; therefore, all staging definitions are according to the 7th edition.^22^,^36^ Finally, even though only (very) high-volume centers were included, the number of DP-CAR procedures per center was very low. We can only speculate that outcomes may improve with higher volumes.

In conclusion, this study showed that DP-CAR with (neo-)adjuvant treatment (82% of the cases) is associated with an acceptable median overall survival of 18 months. Future efforts should be designed to reduce the 90-day mortality to acceptable levels through better patient selection or centralization of treatment.

## Electronic Supplementary Material

Below is the link to the electronic supplementary material.

Supplementary material 1 (DOCX 52 kb)

## References

[1] Bilimoria KY, Bentrem DJ, Ko CY (2007). Validation of the 6th edition AJCC pancreatic cancer staging system: report from the National Cancer Database110. Cancer..

[2] Rombouts SJ, Mungroop TH, Heilmann MN (2016). FOLFIRINOX in locally advanced and metastatic pancreatic cancer: a single centre cohort study. J Cancer..

[3] Suker M, Beumer BR, Sadot E (2016). FOLFIRINOX for locally advanced pancreatic cancer: a systematic review and patient-level meta-analysis. Lancet Oncol..

[4] Appleby LH (1953). The coeliac axis in the expansion of the operation for gastric carcinoma. Cancer..

[5] Nimura Y, Hattory T, Miura K, Nakajima N, Hibi M (1976). Our experience of resection of carcinoma of the body and tail of the pancreas by Appleby’s procedure. Operation..

[6] Katz MH, Marsh R, Herman JM (2013). Borderline resectable pancreatic cancer: need for standardization and methods for optimal clinical trial design. Ann Surg Oncol..

[7] Tempero MA, Malafa MP, Behrman SW (2014). Pancreatic adenocarcinoma, version 2.2014. J Natl Compr Cancer Netw..

[8] Bockhorn M, Uzunoglu FG, Adham M (2014). Borderline resectable pancreatic cancer: a consensus statement by the International Study Group of Pancreatic Surgery (ISGPS). Surgery..

[9] Fishman EK, Al-Hawary M, Francis IR, Merchant NB, Sahani D, Tamm E (2015). NCCN, clinical practice guidelines in oncology: pancreatic adenocarcinoma. version 2.2015. Natl Compr Cancer Netw..

[10] Callery MP, Chang KJ, Fishman EK, Talamonti MS, William Traverso L, Linehan DC (2009). Pretreatment assessment of resectable and borderline resectable pancreatic cancer: expert consensus statement. Ann Surg Oncol..

[11] Varadhachary GR, Tamm EP, Abbruzzese JL (2006). Borderline resectable pancreatic cancer: definitions, management, and role of preoperative therapy. Ann Surg Oncol..

[12] Landelijke-werkgroep-Gastrointestinale-TUMOREN. Pancreatic cancer: Dutch guidelines, version 2.0. Integraal Kankercentrum Nederland; 2011. http://www.oncoline.nl/pancreascarcinoom. Accessed 5 Jan 2015.

[13] Seufferlein T, Bachet JB, Van Cutsem E, Rougier P (2012). Pancreatic adenocarcinoma: ESMO-ESDO clinical practice guidelines for diagnosis, treatment and follow-up. Ann Oncol..

[14] Klompmaker S, De Rooij T, Korteweg JJ (2016). Systematic review of outcomes after distal pancreatectomy with coeliac axis resection for locally advanced pancreatic cancer. Br J Surg..

[15] Kondo S, Katoh H, Shimizu T (2000). Preoperative embolization of the common hepatic artery in preparation for radical pancreatectomy for pancreas body cancer. Hepatogastroenterology..

[16] Conroy T, Desseigne F, Ychou M (2011). FOLFIRINOX versus gemcitabine for metastatic pancreatic cancer. N Engl J Med..

[17] Ocuin LM, Miller-Ocuin JL, Novak SM (2016). Robotic and open distal pancreatectomy with celiac axis resection for locally advanced pancreatic body tumors: a single institutional assessment of perioperative outcomes and survival. HPB..

[18] Peters NA, Javed AA, Cameron JL (2016). Modified Appleby procedure for pancreatic adenocarcinoma: does improved neoadjuvant therapy warrant such an aggressive approach?. Ann Surg Oncol..

[19] Nakamura T, Hirano S, Noji T (2016). Distal pancreatectomy with en bloc celiac axis resection (modified Appleby procedure) for locally advanced pancreatic body cancer: a single-center review of 80 consecutive patients. Ann Surg Oncol..

[20] Sugiura T, Okamura Y, Ito T, Yamamoto Y, Uesaka K (2016). Surgical indications of distal pancreatectomy with celiac axis resection for pancreatic body/tail cancer. World J Surg..

[21] Klompmaker S, van Hilst J, Gerritsen1 S, et al. Pan-European E-AHPBA series of distal pancreatectomy with celiac axis resection (DP-CAR; Appleby) for cancer. 2016. http://www.e-mips.org/wp-content/uploads/sites/3/2016/12/Protocol-EAHPBA-Appleby-cohort-2016.6.3.pdf. Accessed 3 June 2016.

[22] Compton C, Byrd D, Garcia-Aguilar J, Kurtzman S, Olawaiye A, Washington M, Edge S, Byrd D, Compton C, Fritz A, Greene F, Trotti A (2010). Exocrine and endocrine pancreas. AJCC Cancer Staging Manual.

[23] Dindo D, Demartines N, Clavien P-A (2004). Classification of surgical complications: a new proposal with evaluation in a cohort of 6336 patients and results of a survey. Ann Surg..

[24] Wente MN, Bassi C, Dervenis C (2007). Delayed gastric emptying (DGE) after pancreatic surgery: a suggested definition by the International Study Group of Pancreatic Surgery (ISGPS). Surgery..

[25] Wente MN, Veit JA, Bassi C, Dervenis C (2007). Postpancreatectomy hemorrhage (PPH)– an International Study Group of Pancreatic Surgery (ISGPS) definition. Surgery..

[26] Bassi C, Marchegiani G, Dervenis C (2017). The 2016 update of the International Study Group (ISGPS) definition and grading of postoperative pancreatic fistula: 11 years after. Surg..

[27] Mangram AJ, Horan TC, Pearson ML, Silver LC, Jarvis WR (1999). Guideline for Prevention of Surgical Site Infection, 1999. Centers for Disease Control and Prevention (CDC) Hospital Infection Control Practices Advisory Committee. Am J Infect Control..

[28] The Royal College of Pathologists. Standards and minimum datasets for reporting cancers minimum dataset for the histopathological reporting of pancreatic, ampulla of Vater and bile duct carcinoma. London: The Royal College of Pathologists. 2002;261035.

[29] Sadot E, Doussot A, O’Reilly EM (2015). FOLFIRINOX induction therapy for stage 3 pancreatic adenocarcinoma. Ann Surg Oncol..

[30] Conroy T, Paillot B, François E (2005). Irinotecan plus oxaliplatin and leucovorin-modulated fluorouracil in advanced pancreatic cancer—a Groupe Tumeurs Digestives of the Fédération Nationale des Centres de Lutte Contre le Cancer study. J Clin Oncol..

[31] Neoptolemos JP, Palmer DH, Ghaneh P (2017). Comparison of adjuvant gemcitabine and capecitabine with gemcitabine monotherapy in patients with resected pancreatic cancer (ESPAC-4): a multicentre, open-label, randomised, phase 3 trial. Lancet..

[32] Cesaretti M, Abdel-Rehim M, Barbier L, Dokmak S, Hammel P, Sauvanet A (2016). Modified Appleby procedure for borderline resectable/locally advanced distal pancreatic adenocarcinoma: a major procedure for selected patients. J Visc Surg..

[33] Okada K, Kawai M, Tani M (2014). Preservation of the left gastric artery on the basis of anatomical features in patients undergoing distal pancreatectomy with celiac axis en-bloc resection (DP-CAR). World J Surg..

[34] Fusai GK, Pereira S, Valente R, Ravikumar R, Lykoudis P. The arterial study network. 2015. https://www.thearterialstudy.net. Accessed 10 Sept 2015.

[35] Ferrone CR, Marchegiani G, Hong TS (2015). Radiological and surgical implications of neoadjuvant treatment with FOLFIRINOX for locally advanced and borderline resectable pancreatic cancer. Ann Surg..

[36] Amin MB, Edge S, Greene F, Byrd DR, Brookland RK, Washington MK, Gershenwald JE, Compton CC, Hess KR, Sullivan DC, Milburn Jessup J, Brierley JD, Gaspar LE, Richard L, Schi LRM (2017). AJCC cancer staging manual.

