# Supplementary material for: Outcomes After Distal Pancreatectomy with Celiac Axis Resection for Pancreatic Cancer: A Pan-European Retrospective Cohort Study
Source: Ann Surg Oncol. 2018 Mar 12;25(5):1440–7. doi: 10.1245/s10434-018-6391-z (PMC5891548; doi:10.1245/s10434-018-6391-z)
Supplement: Supplementary file 1 — Supplementary material 1 (DOCX 52 kb) [file 10434_2018_6391_MOESM1_ESM.docx]

SUPPLEMENT 1. NEOADJUVANT AND ADJUVANT TREATMENT CHARACTERISTICS

| Baseline | **Neoadjuvant Chemotherapy (n=15)** | | **Neoadjuvant Chemo-radiotherapy (n=19)** | | **Adjuvant Chemotherapy (n=41)** | |
| --- | --- | --- | --- | --- | --- | --- |
| Female sex, No. (%) | 11 | (73.3) | 7 | (36.2) | 22 | (53.7) |
| Age, median (IQR), y | 56 | (52-59) | 65 | (54-66) | 59 | (52-67) |
| Body-mass-index, median (IQR), kg/m2 | 24.2 | (21.4-28.0) | 23.2 | (20.8-25.7) | 23.2 | (22.0-26.3) |
| ASA-classification, No. (%) |  |  |  |  |  |  |
| ASA-1 | 3 | (20.0) | 3 | (15.8) | 7 | (17.1) |
| ASA-2 | 11 | (73.3) | 16 | (84.2) | 33 | (80.4) |
| ASA-3 | 1 | (6.7) | 0 | - | 1 | (2.4) |
| Chemotherapy agents, No. (%) |  |  |  |  |  |  |
| Gemcitabine | 0 | - | 3 | (15.8) | 27 | (65.9) |
| Gemcitabine / Oxaliplatin | 2 | (13.3) | 5 | (26.3) | 1 | (2.4) |
| Gemcitabine / Oxaliplatin / Fluoracil | 0 | - | 2 | (10.5) | 0 | - |
| Gemcitabine / Capecitabine | 0 | - | 2 | (10.5) | 2 | (4.9) |
| Gemcitabine / Cetuximab | 0 | - | 1 | (5.3) | 0 | - |
| Gemcitabine / Fluoracil | 0 | - | 1 | (5.3) | 0 | - |
| Gemcitabine / Docetaxel / Capecitabine | 1 | (6.7) | 0 | - | 0 | - |
| Gemcitabine / Nab-Paclitaxel / Capecitabine | 1 | (6.7) | 0 | - | 0 | - |
| Gemcitabine / Nab-Paclitaxel / FOLFIRINOX | 1 | (6.7) | 0 | - | 0 | - |
| Gemcitabine / Capecitabine / Oxaliplatin | 0 | - | 1 | (5.3) | 0 | - |
| FOLFIRINOX | 6 | (40.0) | 2 | (10.5) | 2 | (4.9) |
| FOLFIRINOX / Capecitabine | 1 | (6.7) | 0 | - | 0 | - |
| FOLFIRINOX / Gemcitabine | 0 | - | 0 | - | 2 | (4.9) |
| Unknown agent | 2 | (13.3) | 2 | (10.5) | 7 | (17.1) |
| Radiotherapy total doses, No. (%) |  |  |  |  |  |  |
| <40 Gy | - | - | 1 | (5.3) | - | - |
| 40-49 Gy | - | - | 5 | (26.3) | - | - |
| 50-59 Gy | - | - | 8 | (42.1) | - | - |
| ≥60 Gy | - | - | 1 | (5.3) | - | - |
| Unknown | - | - | 4 | (21.1) | - | - |
| Cycles of treatment, No. (%) |  |  |  |  |  |  |
| 1-4 | 2 | (13.3) | 2 | (10.5) | 8 | (19.5) |
| 5-9 | 2 | (13.3) | 6 | (31.6) | 12 | (29.3) |
| 10-14 | 4 | (26.7) | 0 | - | 0 | - |
| ≥15 | 1 | (6.7) | 4 | (21.1) | 1 | (2.4) |
| Unknown | 6 | (40.0) | 6 | (31.6) | 20 | (48.8) |

SUPPLEMENT 2. SENSITIVITY ANALYSES FOR 90-DAY MORTALITY

|  |  |  | 90-day mortality | |
| --- | --- | --- | --- | --- |
| **Sub group** | **Total** | **Events** | **Risk Ratio** | **95% Conf. Int.** |
| High- versus low-volume center (PD)* | 43/24 | 5/6 | 0.47 | (0.16-1.37) |
| High- versus low-volume center (DP-CAR) ** | 25/42 | 2/9 | 0.37 | (0.09-1.59) |
| Procedure year '09-'16 vs '00-'08 | 51/16 | 8/3 | 0.84 | (0.25-2.78) |

* Above the median annual case volume of 70

** Above a total case volume of 5

SUPPLEMENT 3A. SURVIVAL AFTER (NEO-)ADJUVANT CHEMOTHERAPY/ CHEMORADIATION AND DP-CAR – INCLUDING 90-DAY SURGICAL MORTALITY

CAPTION: Kaplan-Meier survival curves for all patients (n=68), stratified by neoadjuvant or adjuvant chemotherapy/chemoradiation *versus* no (neo-)adjuvant therapy. This analysis includes 90-day mortality in 3 patients with chemotherapy/chemoradiation and 8 patients without (neo-)adjuvant therapy.

SUPPLEMENT 3B. SURVIVAL AFTER NEOADJUVANT CHEMOTHERAPY/CHEMORADIATION AND DP-CAR – INCLUDING 90-DAY SURGICAL MORTALITY

CAPTION: Kaplan-Meier survival curves for all patients (n=68), stratified by neoadjuvant chemotherapy/ chemoradiation *versus* no neoadjuvant therapy. This analysis includes 90-day mortality in 3 patients with neoadjuvant chemotherapy/chemoradiation and 8 patients without neoadjuvant therapy.

SUPPLEMENT 3c. CONDITIONAL SURVIVAL AFTER DP-CAR AND ADJUVANT CHEMOTHERAPY/ CHEMORADIATION FOR PANCREATIC DUCTAL ADENOCARCINOMA

CAPTION: Kaplan-Meier survival curves stratified by adjuvant chemotherapy/chemoradiation *versus* no adjuvant therapy for all pancreatic ductal adenocarcinoma patients who survived the first 90 days (n=49). Excluded were 2 patients with missing adjuvant chemotherapy data. Median overall survival was 33 *versus* 11 months. We observed no differences in age, preoperative t-stage, major morbidity, R0 resection, and lymph node metastasis between patients who received adjuvant chemotherapy *versus* patients who did not. Vertical bars indicate censored cases.

SUPPLEMENT 4. UNIVARIABLE AND MULTIVARIABLE ANALYSIS FOR 90-DAY MORTALITY

|  | *Univariable analysis* | | | | |  | *Multivariable analysis* | | | | |
| --- | --- | --- | --- | --- | --- | --- | --- | --- | --- | --- | --- |
|  | **Odds Ratio** | **95% Conf. Int.** | | | ***P* Value** |  | **Odds Ratio** | **95% Conf. Int.** | | | ***P* Value** |
| Male sex | 12.40 | 1.49 | - | 103.50 | 0.02 |  | 9.45 | 1.07 | - | 83.09 | 0.04 |
| Age per year | 2.89 | 0.46 | - | 18.17 | 0.26 |  | 1.00 | 0.93 | - | 1.07 | NS |
| Body-mass-index per unit | 0.99 | 0.83 | - | 1.19 | 0.93 |  |  |  | - |  | - |
| ASA-classification ≥ 3 | 1.02 | 0.95 | - | 1.08 | 0.63 |  |  |  | - |  | - |
| Prior surgical history ≥ 1 | 0.77 | 0.18 | - | 3.28 | 0.73 |  |  |  | - |  | - |
| Additional organ involvement* | 2.20 | 0.48 | - | 10.11 | 0.31 |  |  |  | - |  | - |
| Additional vascular involvement* | 0.34 | 0.08 | - | 1.40 | 0.14 |  | 0.37 | 0.07 | - | 1.95 | NS |
| Preoperative tumor size per mm | 1.00 | 0.96 | - | 1.03 | 0.75 |  |  |  | - |  | - |
| *AJCC Staging*** |  |  |  |  |  |  |  |  |  |  | - |
| T-stage ≥ 3 | 0.39 | 0.03 | - | 4.75 | 0.46 |  |  |  | - |  | - |
| N-stage > 0 | 0.35 | 0.07 | - | 1.79 | 0.21 |  |  |  | - |  | - |
| *Neoadjuvant treatment* |  |  |  |  |  |  |  |  |  |  | - |
| Chemotherapy | 0.33 | 0.08 | - | 1.35 | 0.12 |  | 0.57 | 0.11 | - | 2.89 | NS |
| Radiotherapy | 0.56 | 0.11 | - | 2.86 | 0.48 |  |  |  | - |  | - |
| Hepatic artery embolization | 2.34 | 0.58 | - | 9.42 | 0.23 |  |  |  | - |  | - |
| Intent to perform DP-CAR | 0.34 | 0.08 | - | 1.39 | 0.13 |  | 0.44 | 0.07 | - | 2.74 | NS |
| Annual PD case volume ≥ 70 *** | 0.39 | 0.11 | - | 1.47 | 0.17 |  | 0.33 | 0.07 | - | 1.62 | NS |

CAPTION: Abbreviations: PD, pancreatoduodenectomy. * Other than celiac axis, pancreas, or spleen. ** Based on the AJCC criteria.^22^

*** Equal to the 50th percentile.
